# Effects of Increasing Levels of Defatted Rice Bran on Intestinal Physical Barrier and Bacteria in Finishing Pigs

**DOI:** 10.3390/ani9121039

**Published:** 2019-11-28

**Authors:** Huan Wang, Pinghua Li, Taoran Du, Guang Pu, Lijuan Fan, Chen Gao, Peipei Niu, Chengwu Wu, Wuduo Zhou, Ruihua Huang

**Affiliations:** 1 Institute of Swine Science, Nanjing Agricultural University, Nanjing 210095, China; 2 Huaian Academy, Nanjing Agricultural University, Huaian 223003, China; 3 Industrial Technology System Integration Innovation Center of Jiangsu Modern Agriculture (PIG), Nanjing 210095, China; 4 Nanjing Agricultural University’s New Rural Research and Development Corporation of Huaian City, Huaian 223003, China

**Keywords:** defatted rice bran, corns, intestinal barrier, bacteria

## Abstract

**Simple Summary:**

In China, the largest pig-raising country in the world, feed resources are gradually scarce, and the imports of grain crops including corn are increasing. Therefore, it is an urgent problem to find alternatives to grain feed materials. Defatted rice bran (DFRB), an abundant and underutilized agricultural coproduct of brown rice refining process, is rich in energy and dietary fiber (DF). The aims of this study were to assess the effects of increasing levels of DFRB (0%, 7%, 14%, 21%, and 28% DFRB) as a replacement for corns on intestinal physical barrier function and numbers of specific bacteria, and determine the optimal substitution level of DFRB in finishing pigs. We found that 7% DFRB as a replacement for corns had a beneficial effect on intestinal wall thickness, *Bifidobacterium* and *Clostridium perfringens* (*C. perfringens*), and had no adverse effect on intestinal permeability and *Escherichia coli*.

**Abstract:**

The aims of this study were to assess the effects of increasing levels of DFRB as a replacement for corns on intestinal physical barrier function and bacteria of finishing pigs. A total of 35 castrated finishing pigs (age: 158.5 ± 2.0 d, initial body weight: 62.9 ± 0.8 kg) were randomly divided into five dietary treatments (seven replicates/treatment) for a 28-day experimental period, i.e., a control diet with basal diet, and four experimental diets in which maize was replaced by 7%, 14%, 21%, and 28% DFRB, respectively. The results showed that serum endotoxins concentration and diamine oxidase (DAO) activity were both increased (linear, *p* = 0.0004, 0.001, respectively) with DFRB level. However, compared with control group, serum endotoxins concentration and DAO activity were not different in pigs fed with 7% DFRB in the diet. There was a quadratic response in serum D-lactate concentration to the increased DFRB (quadratic, *p* = 0.021). In the cecum, thickness of the intestinal wall significantly increased with increasing levels of DFRB in the diets (linear, *p* = 0.033), while crypt depth/thickness of the intestinal wall ratio significantly decreased with increasing level of DFRB in the diets (linear, *p* = 0.043). In the jejunum, total bacteria, *Escherichia coli*, and *Bifidobacterium* all responded quadratically to increasing levels of DFRB in the diets (quadratic, *p* = 0.003, 0.001, 0.006, respectively). Additionally, there was no difference in *Escherichia coli* in pigs fed 0%, 7%, and 14% DFRB diets. In the colon, there were quadratic responses in *C. perfringens* to the increased DFRB (quadratic, *p* = 0.023). *C. perfringens* reduced as the DFRB concentration increased from 0% to 14% and then increased. When D-lactate, total bacteria, *Escherichia coli*, *Bifidobacterium*, and *C. perfringens* were considered, the optimal substitution level of DFRB were 12.00%, 11.84%, 7.50%, 8.92%, and 15.92%, respectively. In conclusion, 7% DFRB had a beneficial effect on intestinal wall thickness, *Bifidobacterium* and *C. perfringens,* and had no adverse effect on intestinal permeability and *Escherichia coli*.

## 1. Introduction

In China, the largest pig-raising country in the world, feed resources are gradually scarce, and the imports of some grain crops including corns are increasing. During the last decade, the pattern of the feed industry has changed dramatically in price and accessibility of animal feed raw materials [1]. Therefore, it is an urgent problem to find alternatives to grain feed materials. The supply of milling by-products, which is a rich source of dietary fiber (DF), has increased. It is essential to understand the effects of these new and potential DF sources on gut health in pigs. A lot was already known about the impact of DF on swine nutrition [2,3,4,5,6]. In addition, people are increasingly interested in adding dietary fiber to pig diets because DF has been found to modulate gut microbiota and improve gut health [7].

Defatted rice bran (DFRB), an abundant and underutilized agricultural coproduct of the brown rice refining process, is rich in energy and DF [8,9]. The concentration of soluble dietary fiber and insoluble dietary fiber were 1.4% and 26.1% in DFRB, respectively (measured value of the present study). Ingredients of DFRB usually increase beneficial bacteria, reduce potentially pathogenic bacteria populations, and improve gut barrier function [10,11,12,13]. Some studies have indicated that addition of rice bran in diets may have a prebiotic effect. Addition of 10% and 20% full fatted rice bran (FFRB) in mice diets significantly reduced the enteric burden of *Salmonella* infection and increased *Lactobacillus* [13]. Adding 10% rice bran to mice diets increased *Lactobacillus* [10]. Addition of 10% rice bran improved feed utilization efficiency and tended to increase intestinal *Bifidobacteria* in weaning pigs [14]. Arabinoxylan (insoluble dietary fiber) reduced colonic mucosa permeability of healthy humans [11].

However, the high level of DFRB may have a negative effect on growth performance, intestinal bacteria, and gut barrier function because of the high concentration of DF. Warren et al. [15] found addition of 10% or 20% DFRB to growing pigs’ diets had no effect on growth performance, but addition of 30% reduced gain to feed ratio (G:F). Gloria et al. [16] found that average daily feed intake (ADFI) increased linearly and G:F decreased linearly as DFRB increased from 0% to 30% in the diets of finishing pigs. The high mixed-linked β-glucan (soluble dietary fiber) in diets fed to piglets significantly reduced numbers of *Lactobacilli* in the small intestine and decreased the microbial diversity in the colon [17]. Addition of 10% guar gum which is rich in soluble dietary fiber increased colonization of enterotoxigenic *E. coli* and reduced body weight gain in pigs infected with enterotoxigenic *E. coli* [18].

Our previous study has shown that the growth performance of Suhuai finishing pigs were not affected by 28% DFRB [19]. Intestinal barrier function and bacteria are very important to porcine health, whereas effects of increasing levels of DFRB on intestinal barrier function and bacteria was not clear. We hypothesized that moderate levels of DF may improve intestinal barrier function and beneficial bacteria, but excessive levels of DF may have a negative effect on them, and there is an optimal replacement of DFRB for porcine intestinal health.

Therefore, the aims of this study were to assess the effects of increasing levels of DFRB as a replacement for corns on intestinal physical barrier function and bacteria and determine the optimal substitution level of DFRB in finishing pigs.

## 2. Materials and Methods

### 2.1. Experimental Design and Animal Management

A total of 35 Suhuai castrated finishing pigs (age: 158.5 ± 2.0 d, initial body weight: 62.9 ± 0.8 kg) were blocked by initial body weight and randomly divided into one of five experimental diets using a complete randomized block design. Suhuai pig is a new breed that is bred by Chinese Huai pig (25%) and Western Large white pig (75%). The five treatments included a control group and four experimental groups in which maize was separately replaced by 7%, 14%, 21%, and 28% DFRB. All pigs were fed by the Osborne Testing Stations System (OTSS) and it can accurately record daily intake, daily weight gain for each pig. Therefore, each pig was identified as a replicate and there were seven replicates in each treatment. The size of each pen was 5.25 × 2.50 m. The pre-feeding period of the trial was 10 days and all pigs were fed with the same control diet during this period. The feeding experiment lasted 28 d. The finishing body weight of control group, 7%, 14%, 21%, and 28% DFRB group were 82.7 ± 2.1, 82.2 ± 2.0, 84.8 ± 2.1, 83.9 ± 3.0, and 84.8 ± 2.9 kg, respectively. All the pigs were held in the same pigsty with a half seam floor, air-source heat pumps, and a fan system. The temperature inside the piggery varied from 15.5 to 19.6 °C for the whole experimental period. Pigs had *ad libitum* access to feed by OTSS and water by water-saving type stainless-steel drinker. Pigs were healthy and no mortality or diarrhea was observed throughout the experiment. The experimental protocol and procedures were approved by the Animal Care and Use Committee of Nanjing Agricultural University, China (with protocol SYXK (Su) 2017-0007).

### 2.2. Diet Design

The basal diet used for pigs was formulated according to the Feeding Standard of Swine 60–90 kg Standard of Meat-fat Type Growing-finishing Pig (NY/T 65-2004). It was mainly maize that was replaced by 7%, 14%, 21%, and 28% DFRB in four experimental diets. In addition to maize, the other raw materials including wheat bran, soybean oil, lysine also made a small change in four experimental diets to balance metabolic energy and amino acids among five groups. The feed was produced by Huaian Zhengchang Feed Co., Ltd. (Jiangsu, China). Crude protein (CP), crude fiber (CF), ether extract (EE), acid detergent fiber (ADF), neutral detergent fiber (NDF), and hemicellulose contents of corn and DFRB are summarized in Table 1. The chemical characteristics of five diets were analyzed as previously described [20] and presented in Table 2.

### 2.3. Sample Collection

After the 28-d trial, blood samples were collected from jugular vein and centrifuged at 3000× *g* at 4 °C for 10 min to collect serum [21]. The serum was stored at −80 °C until further analyses of endotoxin, diamine oxidase (DAO), and D-lactate. All pigs were stunned by electric shock and slaughtered. The abdomen of each pig was immediately opened, and the jejunum, cecum, and colon were removed. The middle sections (1 cm) of the colon and cecum were collected and then fixed in 4% paraformaldehyde for histological analysis. Mucosal scrapings from the jejunum, ileum, and colon were prepared and stored at −80 °C for measuring 16S rRNA gene copy numbers in bacteria and the mRNA levels of *Caspase 3*, *Bax*, and *Bcl-2L1*.

### 2.4. Histological Measurements

Since fibers are mainly fermented in the large intestine and may have an impact on morphology of the large intestine, we only measured caecal and colonic mucosal morphology. Crypt depth and thickness of the intestinal wall were determined as described by Shen et al. [22]. Fixed intestinal segments were dehydrated with alcohol and encapsulated with paraffin. Consecutive sections at 5 μm thickness were stained with hematoxylin-eosin for histology morphological measurements. The crypt depth was determined on well oriented crypts as distance from the crypt mouth to the crypt base at the basement membrane. Thickness of the intestinal wall was determined as vertical distance from the lateral side of the intestinal wall to the crypt mouth. Crypt depth and thickness of the intestinal wall was measured with a Nikon Eclipse 80i microscope (Nikon Company, Tokyo, Japan).

### 2.5. Blood Sample Analysis

According to the manufacturer’s instructions, the levels of endotoxin, D-lactate, and DAO in the serum were measured by a reagent kit (Jiancheng Bioengineering Institute of Nanjing, Nanjing, Jiangsu, China). The range of the detection of endotoxin, D-lactate, and DAO was 0–1600 EU/L, 0–4.8 μg/L, and 0–80 ng/mL, respectively. Serum was diluted 10 times when D-lactate was measured.

### 2.6. DNA Isolation, Design, and Validation of Primers for Total Bacteria Escherichia coli, Clostridium perfringens, Bifidobacterium and Lactobacillus

Bacterial DNA of the intestinal mucosal scrapings was extracted by FastDNA^®^ Spin Kit for Soil (MP Biomedicals, Irvine, CA, USA). All the primers (Table 3) were commercially synthesized by Beijing Tsingke Biotech Co., Ltd. DNA quality and concentration of were measured using UL-1000 (Shanghai Meixi Instrument Co., Ltd., Shanghai, China). For the quantification of bacteria in samples, standard curves were made by constructing standard plasmids, as described by Han et al. [23]. The specific PCR product of target bacteria was purified by the Cycle Pure Kit PCR (OMEGA Bio-tek, Norcross, GA, USA), and inserted into a Versatile Simple Vector (TsingKe, Nanjing, China). The plasmid DNA was extracted using the AxyPrep^TM^ Plasmid Miniprep Kit (AXYGEN, Fremont, CA, USA) and standard plasmids were constructed successfully. The copies were calculated by the following formula: (6.0233 × 10^23^copies/mol × DNA concentration (μg/mL))/(660 × 10^9^ × DNA size (bp)). A 10-fold serial dilution series of plasmid DNA was used to construct the standard curves for total bacteria, *E. coli*, *C. perfringens*, *Lactobacillus*, and *Bifidobacterium*. Each standard curve was constructed by linear regression of the plotted points, and cycle threshold (CT) values were plotted against the logarithm of template copy numbers. Quantitative analysis of PCR was performed with TB Green ^®^ Premix Ex Taq ™ II (TaRaKa Biotechnology, Shiga, Japan) by an ABI QuantStudio 3 Real-Time PCR System (Applied Biosystems, Foster City, CA, USA).

### 2.7. Real-Time Quantitative PCR

*Caspase-3* is considered to be the most important apoptotic executor, and its activation is a marker of irreversible apoptosis. *Bcl-2L1* protein family plays an important role in regulating apoptosis, in which *Bcl2* is an anti-apoptotic gene and *Bax* is a pro-apoptotic gene. All of them play an important role in the process of apoptosis. To evaluate the effects of increasing levels of DFRB as a replacement for corns on intestinal cell proliferation and apoptosis of finishing pigs, the mRNA levels of *Caspase 3*, *Bax*, and *Bcl-2L1* were detected by real-time quantitative PCR.

According to the manufacturer’s guidelines, total RNA of intestinal mucosa was extracted using TRIZOL (Shanghai Yuanye Biotechnology Co., Ltd., Shanghai, China). The RNA concentration and quality were measured using UL-1000 (Shanghai Meixi Instrument Co., Ltd., Shanghai, China). The RNA samples were reverse transcribed into complementary DNA using 5X All-In-One RT MasterMix (Applied Biological Materials, Richmond, B.C., Canada). Quantitative analysis of PCR was performed with TB Green ^®^ Premix Ex Taq ™ II (TaRaKa, Shiga, Japan) by an ABI QuantStudio 3 Real-Time PCR System (Applied Biosystems, Foster City, CA, USA).

The reaction was performed using the following cycle program: a hold stage at 95 °C for 10 min; 35 cycles for PCR stage at 95 °C for 15 s and at 60 °C for 60 s; a melt curve stage at 95 °C for 15 s, at 60 °C for 60 s, and at 95 °C for 1 s. All samples were analyzed for three repetitions. The relative expression of the *Caspase 3*, *Bax*, and *Bcl-2L1* mRNA was calculated using the 2^−ΔΔCt^ method [31].

### 2.8. Statistical Analysis

Bacterial 16S rRNA gene copy numbers were transformed (log 10) before statistical analysis. Linear and quadratic effects of dietary treatments on all indices were determined by curve estimation and some indices including serum endotoxins, DAO and *Escherichia coli* in the jejunum in Appendix A Table A1 were also analyzed by one-way ANOVA (SPSS 25.0) (SPSS Inc., Chicago, IL, USA). DFRB was the main effect. Each pig was considered as the experimental unit for all analyses. The α-level was set as 0.05 for significance determination. Data are presented as means with their pooled standard errors. The linear effects was determined by equation:

y = a + bx,(1)

where y is dependent variable, and x is the content of DFRB.

The optimal substitution level of corn by DFRB was predicted by quadratic regression equation as described by Souza et al. [32]. Quadratic regression equation:

y = a + bx + cx^2(2)

the optimal substitution level (%) = −b/2 × c(3)

where y is dependent variable, and x is the content of DFRB.

## 3. Results

### 3.1. Effects of Varying DFRB Levels on Intestinal Permeability

Serum endotoxins, D-lactate concentration, and DAO activity are shown in Table 4. Serum endotoxins concentration and DAO activity were both increased (linear, *p* = 0.0004, 0.001, respectively) as the DFRB content of the diets increased. However, compared with control group, serum endotoxins concentration and DAO activity were not different in pigs fed with 7% DFRB in the diet (Appendix A Table A1). There was a quadratic response in serum D-lactate concentration to the increased DFRB (quadratic, *p* = 0.021).

### 3.2. Effect of Varying DFRB Levels on Intestinal Morphology

We observed the effects of varying DFRB levels on intestinal morphology (Table 5). The thickness of the intestinal wall significantly increased with the increasing level of DFRB in the diets (linear, *p* = 0.033), while crypt depth/thickness of the intestinal wall ratio significantly decreased with the increasing level of DFRB in the diets (linear, *p* = 0.043) in the cecum. Crypt depth was not influenced by the level of DFRB in the diet in the cecum. Moreover, the crypt depth, thickness of the intestinal wall, and crypt depth/thickness of the intestinal wall ratio in the colon were not influenced by the level of DFRB in the diet.

### 3.3. Effect of Varying DFRB Levels on 16S rRNA Gene Copy numbers in Bacteria

The effects of varying DFRB levels on 16S rRNA gene copy numbers in bacteria are reported in Table 6. In the jejunum, total bacteria, *Escherichia coli*, and *Bifidobacterium* all responded quadratically to increasing levels of DFRB in the diets (quadratic, *p* = 0.003, 0.001, 0.006, respectively) while *C. perfringens* and *Lactobacillus* were not influenced by the level of DFRB in the diets. There was no difference in *Escherichia coli* in pigs fed 0%, 7%, and 14% DFRB diets (Appendix A Table A1). In the ileum, total bacteria, *Escherichia coli*, *C. perfringens*, *Lactobacillus*, and *Bifidobacterium* were not influenced by the level of DFRB in the diets. In the colon, there were quadratic responses in *C. perfringens* to the increased DFRB (quadratic, *p* = 0.023). *C. perfringens* reduced as the DFRB concentration increased from 0% to 14% and then increased as the DFRB concentration increased from 14% to 28%.

### 3.4. Effect of Varying DFRB Levels on Intestinal Gene Expression

The mRNA levels of *Caspase 3*, *Bax*, and *Bcl-2L1* were not influenced by the level of DFRB in the diets in jejunum, ileum, and colon.

### 3.5. Calculation of the Optimal Substitution Level of DFRB

The result of the calculation of the optimal substitution level of DFRB for corns is shown in Table 7. In this experiment, five indicators including D-lactate, total bacteria, *Escherichia coli*, *Bifidobacterium*, and *C. perfringens* were quadratically correlated with the level of DFRB. They were used to calculate the optimal substitution level of DFRB for maize. When D-lactate, total bacteria, *Escherichia coli*, *Bifidobacterium*, and *C. perfringens* were considered, the optimal substitution level of DFRB was 12.00%, 11.84%, 7.50%, 8.92%, and 15.92%, respectively.

## 4. Discussion

Integrated intestinal mucosal barrier is important for the defense of pathogenic bacteria [33,34]. The intestinal permeability can be increased by the injured intestinal mucosal barrier. The intestinal barrier function has to do with many factors, including endotoxins, D-lactate concentration, and DAO activity in serum [35,36,37,38]. They have been considered as markers for evaluating the extent of intestinal mucosal damage and repair [39]. As one of the secretions of *Escherichia coli*, serum endotoxins activity increased with increased intestinal permeability or injury to intestinal barrier integrity [40]. D-Lactate is the end product of intestinal bacteria. Mammals produced neither D-lactate nor D-lactate dehydrogenase. Hence, they maintain a lower level of D-Lactate in healthy conditions [41]. When intestinal mucosal integrity is impaired, almost all D-lactate will release into the blood. Thus, this indicates that serum D-Lactate reflects the integrity and maturity of intestinal mucosa [42]. DAO is one of the DAO catalyzed by deaminases, only exists in the villi of the upper small intestine, and its increasing concentrations indicate increased intestinal epithelial permeability or damage to intestinal barrier function [43,44]. Serum endotoxins concentration and DAO activity were both increased with the DFRB level. There was a quadratic response in serum D-lactate concentration to the increased DFRB. However, compared with control group, serum endotoxins concentration and DAO activity were not different in pigs fed with 7% DFRB in the diet. Therefore, 7% DFRB had had no adverse effect on intestinal permeability.

On the other hand, integral morphological structure is important for the intestinal tract to maintain the ability to secret, digest, and absorb nutrients. As fibers are mainly fermented in the large intestine and may have an impact on morphology of the large intestine, we only measured cecal and colonic mucosal morphology. The present study showed that colonic morphology was not influenced by the increased DFRB. However, in the cecum, thickness of the intestinal wall significantly increased with increasing level of DFRB in the diets while crypt depth/thickness of the intestinal wall ratio decreased with the increasing level of DFRB in the diets. One possible explanation for this result is that muscle thickness increased but crypt depth remained unchanged with the increasing level of DFRB in the diets. Additionally, increased muscle thickness may increase the mixing of intestinal contents to increase digestibility of the DF.

The intestinal microflora is very important to intestinal health, not only because the imbalance of the microflora may lead to an inflammation response, but also may compete with the host for nutrients [45]. The microflora enhances the intestinal mucosa barrier function and reduces the adhesion of pathogenic microorganisms to the mucosa, thus reducing the chance of pathogenic microorganisms entering enterocytes. *Lactobacillus* and *Bifidobacteria* are the main beneficial bacteria in the intestinal tract of mammals. They play a resistant role against intestinal pathogens through a variety of mechanisms. *Lactobacillus* is considered to be a reflection of changes in the population structure of beneficial bacteria because a large number of intestinal bacteria cannot be cultured [46]. It is controversial to consider *E. coli* as a marker of pathogenic bacteria; however, the number of *E. coli* increased in the intestinal tract of diarrhea pigs [47]. Therefore, the reduction of *E. coli* caused by dietary intervention is considered to be beneficial by many people to a certain extent [48,49,50]. Many studies have shown that DF is beneficial to the proliferation of beneficial bacteria and inhibits harmful bacteria. Drew et al. [51] found that wheat-based diets increased *Bifidobacterium* and reduced total aerobes and *Clostridium*. Similarly, Nielsen et al. [52] reported that addition of arabinoxylan (AX) in pig diets increased *Bifidobacterium* and *Lactobacillus* in the feces. In line with the previous findings, this study shows that there were declined *Escherichia coli* and increased *Bifidobacterium* as DFRB increased from 0% to 7%. *Escherichia coli* increased and *Bifidobacterium* declined when 14% DFRB was fed. One possible explanation might be that high-fiber diets have a negative effect on intestinal barrier function and is not conducive to the growth of beneficial bacteria. The high level of DFRB have a negative effect on the intestinal barrier function, which may be mediated by the changes of microbial composition and the accompanying changes of intestinal permeability.

This study shows the mRNA levels of *Caspase 3*, *Bax*, and *Bcl-2L1* were not influenced by the level of DFRB in the diets in jejunum, ileum, and colon. The results showed that the addition of defatted rice bran in the diet did not affect the apoptotic process of intestinal cells. This is consistent with the previous study made by Gregoire et al. [53]. They reported that a short-term increase in dietary fiber does not result in a significant difference in cell proliferation [53]. However, Jin et al. [54] found that the number of epithelial cells exhibiting DNA fragmentation (indicating programmed cell death) was greater in growing pigs consuming the high-fiber diet than in the low-fiber diet group for jejunum and ileum. The difference in the results may be due to the different types of fibers and the different stages of animal growth.

## 5. Conclusions

In conclusion, 7% DFRB had a beneficial effect on intestinal wall thickness, *Bifidobacterium*, and *C. perfringens*, and had no adverse effect on intestinal permeability and *Escherichia coli*.

## Figures and Tables

**Table 1:** Nutrient level of corn and DFRB.

Ingredients (%)	Corn	DFRB
CP	10.62	18.54
CF	2.42	10.88
EE	5.49	2.90
ADF	3.24	11.39
NDF	11.12	30.09
Hemicellulose ^1^	7.82	18.44

CP, Crude protein; CF, crude fiber; EE, ether extract; ADF, acid detergent fiber; NDF, neutral detergent fiber; DFRB, defatted rice bran; ^1^ Hemicellulose = NDF − ADF.

**Table 2:** Ingredients and nutrient level of the experimental diets.

Items	Diet				
	Control	7%	14%	21%	28%
Ingredients (%)					
Corns	68.61	62.00	55.00	48.00	41.00
Wheat bran	15.40	15.80	16.15	16.67	17.21
DFRB	0.00	7.00	14.00	21.00	28.00
Soybean meal	13.30	11.70	10.40	8.95	7.50
Soybean oil	0.00	0.84	1.83	2.78	3.74
98.5% Lysine	0.03	0.04	0.03	0.03	0.03
Salt	0.30	0.30	0.30	0.30	0.30
Limestone	0.82	0.85	0.85	0.85	0.85
CaHPO_4_	0.75	0.68	0.65	0.63	0.58
60% Choline	0.04	0.04	0.04	0.04	0.04
Premix ^1^	0.40	0.40	0.40	0.40	0.40
Nutrient level ^2^					
DM (%)	88.56	88.68	88.93	89.16	88.46
Digestible energy/(MJ·kg^−1^)	13.13	13.13	13.13	13.13	13.13
CP (%)	15.60	16.67	16.13	15.73	16.40
CF (%)	8.89	11.80	12.93	14.35	17.94
Calcium (%)	0.55	0.55	0.55	0.55	0.55
Available phosphorus (%)	0.27	0.27	0.27	0.27	0.27
L-lysine (%)	0.65	0.65	0.65	0.66	0.65
Methionine + cystine (%)	0.45	0.45	0.46	0.47	0.47
IDF (%)	16.14	17.19	18.42	19.32	23.37
SDF (%)	0.52	0.56	0.68	0.73	0.82
TDF (%)	16.70	17.75	19.10	20.05	24.11
ADF (%)	5.53	6.25	6.53	7.08	8.13
NDF (%)	8.89	11.80	12.93	14.35	17.94
EE (%)	5.19	5.08	5.32	5.27	5.38
Hemicellulose (%)	3.80	5.69	7.09	8.00	10.34
Cellulose (%)	4.06	4.43	4.71	5.09	5.79
Lignin (%)	0.46	0.54	0.72	0.96	1.13

^1^ The premix provided the following per kg of diets: vitamin A 8000 IU, vitamin E 100 mg, vitamin K_3_ 4 mg, vitamin D_3_ 1500 IU, vitamin B_1_ 2 mg, vitamin B_2_ 8 mg, vitamin B_6_ 3 mg, vitamin B_12_ 0.04 mg, niacin 30 mg, Choline 150 mg, biotin 0.13 mg, folic acid 0.6 mg, pantothenic acid 35 mg, Fe 60 mg, Cu 5 mg, Zn 60 mg, Mn 10 mg, Se 0.15 mg, I 0.1 mg. ^2^ DM, dry matter, CP, crude protein, CF, crude fiber, IDF, insoluble dietary fiber, SDF, soluble dietary fiber, TDF, total dietary fiber, EE, ether extract, ADF, acid detergent fiber, NDF, neutral detergent fiber, DFRB, defatted rice bran. DM, CP, CF, IDF, SDF, ADF, NDF, EE, hemicellulose, cellulose and lignin were measured values, while the other nutrient levels were calculated values.

**Table 3:** Primers used for real-time PCR.

Target	Primer Sequence (5′-3′)	Reference
Total bacteria		
Forward	ACTCCTACGGGAGGCAGCAG	[24]
Reverse	ATTACCGCGGCTGCTGG	
*Escherichia coli*		
Forward	CATGCCGCGTGTATGAAGAA	[25]
Reverse	CGGGTAACGTCAATGAGCAAA	
*C. perfringens*		
Forward	CGCATAACGTTGAAAGATGG	[26]
Reverse	CCTTGGTAGGCCGTTACCC	
*Lactobacillus*		
Forward	GCAGCAGTAGGGAATCTTCCA	[27]
Reverse	GCATTYCACCGCTACACATG	
*Bifidobacterium*		
Forward	CGGGTGAGTAATGCGTGACC	[28]
Reverse	TGATAGGACGCGACCCCA	
*GAPDH*		
Forward	GTCGGAGTGAACGGATTTGG	[29]
Reverse	CAATGTCCACTTTGCCAGAGTTAA	
*Bcl-2L1*		
Forward	TGAATCAGAAGCGGAAACCC	[30]
Reverse	GCTCTAGGTGGTCATTCAGGTAAG	
*Bax*		
Forward	AAGCGCATTGGAGATGAACT	[30]
Reverse	CGATCTCGAAGGAAGTCCAG	
*Caspase 3*		
Forward	ACACGCCATGTCATCTTCAGTCC	[30]
Reverse	TTCATAATTCAGGCCTGCCGAG	

**Table 4:** Effects of varying defatted rice bran levels on intestinal permeability ^1^.

Item	Diet					SEM	*p* Value	
	Basal	7%	14%	21%	28%		Linear	Quadratic
Endotoxins, EU/L	219.58	240.42	335.60	421.78	352.86	18.01	<0.001	<0.001
D-lactate, μg/L	4.86	4.00	4.42	4.89	5.15	0.36	0.102	0.021
DAO, ng/mL ^2^	23.38	27.39	23.71	35.16	32.23	1.16	0.001	0.005

^1^ Values are means and pooled SEMs, n = 7. ^2^ DAO, diamine oxidase; SEM, standard error of mean.

**Table 5:** Effect of varying defatted rice bran levels on intestinal morphology.

Item	Diet					SEM	*p* Value	
	Basal	7%	14%	21%	28%		Linear	Quadratic
Cecum								
Crypt depth, μm	452.03	450.95	462.07	434.32	448.66	22.13	0.631	0.884
Thickness of the intestinal wall, μm	1253.06	1319.43	1348.21	1409.07	1336.48	30.12	0.033	0.021
CD/IWT ^1^	0.36	0.34	0.34	0.31	0.34	0.01	0.043	0.063
Colon								
Crypt depth, μm	420.31	431.61	355.04	418.50	405.18	29.73	0.546	0.470
Thickness of the intestinal wall, μm	1115.69	1187.08	1425.81	1130.41	1262.36	28.79	0.251	0.116
CD/IWT	0.38	0.37	0.25	0.37	0.32	0.12	0.210	0.127

^1^ CD/IWT, Crypt depth/thickness of the intestinal wall ratio. Values are means and pooled SEMs, n = 7. SEM, standard error of mean.

**Table 6:** Effect of varying defatted rice bran levels on 16S rRNA gene copy numbers in bacteria, lg (copies/g) ^1^.

Item	Diet					SEM	*p* Value	
	Basal	7%	14%	21%	28%		Linear	Quadratic
Jejunum								
Total bacteria	6.84	6.51	6.49	6.59	7.27	0.17	0.140	0.003
*Escherichia coli*	5.17	4.00	5.08	6.20	6.58	0.54	0.002	0.001
*C. perfringens*	2.10	1.78	1.52	2.03	1.95	0.16	0.810	0.156
*Lactobacillus*	5.53	6.39	4.35	4.96	5.39	0.49	0.379	0.402
*Bifidobacterium*	4.88	5.02	4.82	4.91	4.44	0.10	0.014	0.006
Ileum								
Total bacteria	8.53	7.29	8.43	8.15	8.37	0.13	0.987	0.647
*Escherichia coli*	6.29	5.40	6.02	4.86	6.29	0.14	0.559	0.079
*C. perfringens*	3.15	2.70	3.01	2.43	3.14	0.12	0.509	0.086
*Lactobacillus*	7.19	5.37	7.30	5.76	6.75	0.18	0.394	0.509
*Bifidobacterium*	6.39	5.29	6.35	5.24	6.34	0.11	0.619	0.163
Colon								
Total bacteria	8.33	7.60	8.08	8.01	7.76	0.13	0.160	0.303
*Escherichia coli*	5.75	5.39	5.60	4.92	5.13	0.33	0.123	0.305
*C. perfringens*	4.05	3.41	2.98	3.36	3.63	0.24	0.277	0.023
*Lactobacillus*	6.97	5.39	6.51	6.77	6.31	0.48	0.956	0.482
*Bifidobacterium*	5.01	5.27	5.00	4.83	5.08	0.12	0.437	0.741

^1^ Values are means and pooled SEMs, n = 7. SEM, standard error of mean.

**Table 7:** The optimal substitution level of corns by defatted rice bran in Suhuai finishing pigs.

Item	a ^1^	b	c	Inflexion Point (%)
D-lactate	4.697	−0.072	0.003	12.00
Total bacteria	6.854	−0.073	0.003	11.84
*Escherichia coli*	4.931	−0.105	0.007	7.50
*Bifidobacterium*	4.878	0.023	−0.001	8.92
*C. perfringens*	4.045	−0.121	0.004	15.92

^1^ a–c are coefficients of constant term, primary term and quadratic term, respectively.

## Appendix A

**Table A1:** The results of one-way ANOVA of serum endotoxins, diamine oxidase (DAO), and *Escherichia coli* in the jejunum ^1^.

Item	Diet					SEM	*p* Value
	Basal	7%	14%	21%	28%		ANOVA
Endotoxins, EU/L	219.58 ^c^	240.42 ^c^	335.60 ^b^	421.78 ^a^	352.86 ^b^	18.01	<0.001
DAO ^2^, ng/mL	23.38 ^b^	27.39 ^ab^	23.71 ^b^	35.16 ^a^	32.23 ^a^	1.16	0.001
*Escherichia coli* (Jejunum)	5.17 ^ab^	4.00 ^b^	5.08 ^b^	6.20 ^ab^	7.58 ^a^	0.54	0.006

^1^ Means with similar lowercase letters (a–c) within a row are the same (*p* < 0.05). Values are means and pooled SEMs, n = 7. SEM, standard error of mean. ^2^ DAO, diamine oxidase.
